# Genetics and Learning: How the Genes Influence Educational Attainment

**DOI:** 10.3389/fpsyg.2019.01622

**Published:** 2019-07-10

**Authors:** David Bueno

**Affiliations:** Biomedical, Evolutionary, and Developmental Genetics Section, Faculty of Biology, University of Barcelona, Barcelona, Spain

**Keywords:** genetics, epigenetics, heritability, environment, learning, adaptation

## Abstract

The brain is the organ of thought. The word *thought* is defined as the act of thinking about or considering something: an idea or opinion, or a set of ideas about a particular subject. It implicitly includes the processes of learning. Mental functions, including most if not all aspects of human behavior, such as those related to learning, arise from the activity of the brain. Neural connections that generate and support mental functions are formed throughout life, which enables lifelong learning of new concepts and skills. Both brain formation and function, as well as neural plasticity, are influenced by the activity of a variety of genes and also by epigenetic modifications, which contribute to the regulation of gene expression by adapting it to environmental conditions. In this review, aimed especially at education professionals, I discuss the genetic and epigenetic contributions to mental aspects related to learning processes in terms of heritability. I will argue that, despite most if not all aspects related to learning having a clear genetic background, innate abilities can be enhanced or diminished through educational processes. Thus, the importance of education, in the context of the inheritability of learning processes, will be discussed. The conclusion I draw is that, despite the relatively high genetic heritability shown in most brain processes associated with learning, educational practices are a key contributor to student development, allowing genetically based skills to be enhanced or alternatively diminished. Therefore one of the main goals of education in a changing an uncertain world should be to form adaptable and versatile people who can, and want to, make the most of their capabilities. Thus, knowledge derived from genetics and epigenetics, as well as from neuroscience, should be used to enhance education professionals’ understanding of the biological origins of differences in mental capabilities, thereby empowering them with the possibility to adopt more respectful and flexible educational practices to attain the goal mentioned above.

## Introduction

Despite the fact that learning is a complex task which involves many cognitive functions, the brain is capable of learning new skills and concepts throughout life, interacting dynamically with the environment. Biologically, learning capacity allows behavioral responses to adapt progressively, modifying aspects that may favor the survival of the individual in a complex, dynamic and changing environment. Education relies on this learning capacity and it should optimize cognitive functions taking into account human culture. That is, depending on the specific historical and social context, education should contribute to forming people who are able to transform themselves through self-directed active learning throughout their lives. Such educated people should also be predisposed to enhance their abilities and knowledge in whatever direction they choose, to the limits of possibility, thus growing intellectually and emotionally. In other words, these are people who can, and want to, make the most of their capabilities.

The brain forms during embryonic and fetal development under the direction of certain genetic programs (see Brown et al., 2001, for a general description of brain development). However, it continues building and rebuilding its connectome, i.e., the map of neural connections, throughout the whole of life, thanks to its ability to establish new neural connections. This process of neuronal plasticity is the cellular basis of learning. From the psychological perspective, the ability to learn requires many different cognitive functions: mental abilities that are used in the process of acquiring knowledge. Cognitive functions, such as working memory, cognitive control, attention, intelligence and executive functions, as well as other related functions such as motivation and resilience, develop through the activity of the brain, which in turn depends on neural connectivity and function. Both the construction of the brain and the functioning of its neurons rely on genetic programs, so genes must at least exert some influence on the cognitive functions involved in learning processes.

The human genome is made up of some 20,300 genes (Salzberg, 2018), all of which may present genetic variants, or alleles. The different nucleotide sequences that alleles contain imply differences in the message they encode, which in turn are reflected in the biological functions they control or influence by synthesizing the corresponding proteins. Just as some people have green eyes while others have brown or blue eyes, or that some are taller than others, individual variations due to genetic influences can also be far-reaching and can be found not only in physical traits, but also in psychological ones, including those related to cognitive functions. For example, Davies et al. (2018) identified 148 novel independent genes, on top of the total of 709 previously identified, associated with general cognitive functions; meanwhile, Zwir et al. (2018b) identified 736 genes that are significantly associated with temperament.

To give another example, several genes whose alleles differently influence working memory have also been reported (Karlsgodt et al., 2011). Working memory is a cognitive system responsible for temporarily holding information available for processing. It is included in the so-called executive functions: the set of cognitive processes that are necessary for the cognitive control of behavior. For example, the COMT gene, encoding the enzyme responsible for recycling neurotransmitters such as dopamine (which in turn is associated with motivation and reward in the brain, among other functions), exhibits an allelic system known as Val158Met which appears to influence working memory and educational attainment (Enoch et al., 2009). The nomenclature adopted here refers to the position of the amino acid of the protein encoded by the gene showing allelism and its alternatives, i.e., the amino acid in position 158 of the COMT enzyme could be either a valine (Val) or a methionine (Met), depending on the sequence of the allele. In this context, the Val form could induce lower activity in the prefrontal lobe of the brain, where the neuronal circuits involved in working memory are found, thus causing this cognitive function to be less efficient, on average, in people who carry this allele. In other work, Walter et al. (2011) found that this same allelic system also influences social facilitation: a cognitive feature defined as an improvement in performance produced by the mere presence of others, and which is also important in learning processes.

Despite the existence of genetic influences on most, if not all, cognitive functions associated with learning, there is no doubt that in aspects related to mental life, certain factors make the precise identification and the particular influence of each specific gene and of each particular allele on the many aspects related to learning, very difficult. These factors include: (1) the high number of genes involved, (2) the usually complex interactions between genes, (3) the fact that any gene, and even any allelic system, may influence different psychological domains (for example, working memory and social facilitation, as reported for the Val158Met allelic system of the COMT gene), and (4) the influence of many environmental aspects on mental functions, including social, familiar and educational environments. For example, dozens of different genes have been identified influencing intelligence, as measured by IQ; however, none of these contributes to more than 1% of the total measure of this feature (reviewed by Plomin and von Stumm, 2018). Consequently, the most informative data, from the educational perspective, come from so-called heritability. Heritability is a statistic that, as commonly interpreted, captures how much of the variation of a trait is due to genetic differences. (It does not, however, capture how many genes or which genes/alleles are involved, or how much of the trait relies exclusively on the genome).

This review focuses on the heritability of cognitive functions that are relevant for learning. Children are not a simple *tabula rasa*, as once thought, since they are conditioned by their genome to an extent. The environment, including the educational one, is also a significant factor which can allow them to make the most of their capabilities, including intellectual and emotional aspects. In other words, although the brain is malleable and can be changed through education and daily experiences, and thus so can cognitive functions, its formation and functioning are based on a genetic substrate that influences it to a moderate or high degree. Therefore, knowledge of the existence of genetic and epigenetic influences on the development of cognitive functions and the extent of their influence, may empower education professionals to work toward more respectful and flexible practices. These would take into account the genetic influences, emphasizing the importance of educational practices as an environmental factor that contributes to the potential maximization of students’ skills. What we cannot yet say with a sufficient level of confidence is which traits can be improved more easily or which are difficult to change. Not every trait is equally amenable to meaningful change through educational intervention, as they are influenced by a wide variety of factors, from the range of different genes that may act on a single trait to the concurrence of a large number of environmental factors, including educational ones but also individual experiences that are almost impossible to quantify.

## Understanding Heritability

There is a large body of evidence that supports the conclusion that individual differences in most, if not all, reliable measures of psychological traits, are substantively influenced by genetic factors. Put concisely, psychological traits, including cognitive functions that are indispensable for learning, are partially heritable. To quantify how much of the variation of a trait that is found among people in a given population is due to genetic differences, we use the heritability statistic. Despite its broad use, however, this measure may be easily misinterpreted, leading to misconceptions that are detrimental when it comes to considering educational and psychological backgrounds. First, heritability is not a property of any individual or specific gene, but a parameter of a population. Thus, like other population parameters such as arithmetic mean, it can only be used to describe the phenomenon and its relations or effects at the population level.

Technically, heritability could be defined as the proportion of the variability in any observable characteristic that is associated with genetic variation in the population. That variation implies the existence of alleles contributing differently to the characteristic analyzed. If this reasoning is applied to the variation of cognitive functions among the individuals in a given population, then it can be said that heritability refers to the proportion of the variation of a particular trait that is associated with the genetic variation within that population. Heritability is a measure of what is associated with variation in the trait, but it is not a measure of what causes the trait. It says nothing about the number of genes involved, or the relations between those genes, or which particular genes or alleles are involved, or how much of the trait relies exclusively on the genome.

In order to better understand the concept of the heritability of cognitive functions it is necessary first to comprehend the concept of individual differences. An observable characteristic of an individual is known as their “phenotype”: a broad term for any characteristic that can be observed, measured and analyzed. In this respect, cognitive functions can be considered as phenotypes, as they can be observed, analyzed and measured through appropriate tests. The expression of phenotypes depends on the genetic background of the individual, their so-called genotype, as well as on a number of environmental influences that modulate gene function. The basic assumption here is that phenotypic variance in the population may be divided into genetic and environmental variances. Variance is a statistic that measures how spread out a set of values are from their mean. This assumption can be represented linearly as: V_P_ = V_G_ + V_E_ + V_I_, where V_P_, V_G_ and V_E_ represent phenotypic, genetic and environmental variances, respectively; and V_I_, the variance of the interaction between genes and environment. It is interesting to note that the manifestation of a particular trait, the phenotype, not only depends on genetic background and environment, but also on the interaction between them. Some phenotypes, for example, blood group in the ABO system, only show genetic variance, with no environmental influences. Others, like height, are influenced by genetic, environmental and also interactive variances.

Individual genetic effects in a population may be combined in additive or in non-additive ways. An additive genetic effect is the linear combination of the individual genetic effects. Non-additive genetic effects include different non-linear combinations, for example, the dominance of some alleles over others and epistatic interactions in which the effect of one gene depends on the presence of one or more modifier genes. The difference between additive and non-additive genetic effects differentiates two different concepts of heritability. Broad-sense heritability, which is represented as *H^2^*, considers all genetic effects, while narrow-sense heritability, or *h^2^*, only includes additive genetic affects. From a practical point of view, additive genetic effects are more predictable than non-additive.

Heritability can be estimated using a number of methods. These include twin, family and adoption studies, in addition to or in combination with, molecular genomic analysis either of particular genes or via genome-wide association studies (GWASs), which allow us to associate the phenotype of interest with the genetic sequence of individuals. GWASs are observational studies of a genome-wide set of alleles in different individuals to see if any variant is associated with the trait analyzed. GWASs typically focus on associations between different single-nucleotide polymorphisms (SNPs), which are the most common type of genetic variation among people. Each SNP represents a difference in a single nucleotide within the DNA molecule. There are roughly 4–5 million SNPs in a person’s genome, which may be unique or occur in many individuals. In the context of heritability calculations for cognitive functions, SNPs can be used as biological markers or they may play a direct role in the characteristic analyzed. Heritability captures how much of the variation of a trait between individuals within a population is due to genetic differences, and as mentioned, it must be used only as a parameter of the population.

Classic twin studies compare the similarity of identical (monozygotic) and fraternal (dizygotic) twins reared together, or alternatively, reared apart. Identical twins are genetically identical, while fraternal twins share, on average, 50% of their genes. In fraternal twins reared together, the observed differences in the phenotype analyzed could be attributed either to genetic differences, or to non-shared environmental differences (see below for a discussion of environmental differences that are not shared). Conversely, in identical twins reared apart, phenotypic differences could be attributed solely to non-shared environmental differences. Similarly, family and adoption studies investigating the similarity of different family members combine different degrees of genetic and environmental sharing. For example, Plomin and Spinath (2004) examined correlations of IQ heritability in monozygotic and dizygotic twins, as well as in adopted children. As expected, the correlation is higher in monozygotic (>0.8) than in dizygotic twins (≈0.6). Interestingly, differences between these correlations for intelligence increase during development, suggesting that environmental influences are less relevant, thus making heritability to increase. The correlation in adopted children is around 0.0. To be statistically significant, these studies must rely on a sufficient number of individuals, but the precise number depends on the effect to be detected, from only some tens of pairs to as many as 10,000 individuals for very small effects of some variations (Posthuma and Boomsma, 2000).

Heritability is expressed on a scale ranging from 0 to 1 or alternatively as a percentage, i.e., from 0 to 100%. A value of 0.0 (or 0%) must be interpreted as a trait in which the observed differences are not associated with genetic variation at all, but only with environmental differences. Conversely, a value of 1.0 (or 100%) must be interpreted as a trait in which the observed differences are solely associated with genetic variation: not with environmental differences at all. This latter case does not mean that environment is not important, but that it does not influence the variance. We must take into account that heritability is not a measure of how sensitive a trait might be to a change in environment, but it is a statistic that is heavily dependent on environmental conditions. For example, a trait may have complete heritability (1.0) under specific environmental conditions, yet be altered drastically by environmental changes. This can be seen, for example, in certain metabolic genetic disorders, such as phenylketonuria (PKU), which is due to a mutation in a single gene (the phenylalanine hydroxylase gene). PKU leads to intellectual disability due to the accumulation of the amino acid phenylalanine, among other phenotypic effects (reviewed by Al Hafid and Christodoulou, 2015). Under conditions of normal food intake, PKU has a heritability of 1.0, but dietary interventions that reduce phenylalanine intake from birth make phenotypical consequences negligible. In other words, if conditions changed, the heritability would also change. By increasing environmental variation, the proportion of the phenotypic variance due to genetic diversity would be reduced.

Similarly, and to cite another example in an educational context, Colodro-Conde et al. (2015) reported a shift in the heritability of educational attainment in an Spanish cohort after the introduction of a specific educational policy in 1970, which was intended to promote educational. The shared-environmental variance decreased, leading to an increase in heritability from 0.44 to 0.67 for males, thus supporting the role of educational policy in affecting the relative weight of genetic and environmental factors on educational attainment. Thus, in this way, to say that a specific cognitive skill, for example, resilience, has a heritability of 0.52 in males (Boardman et al., 2008), does not imply that the education received can only affect 0.48 (or 48%) of total differences. Rather, a heritability of 0.52 means that 52% of the variability in resilience responses is associated with the genetic variation in the actual population: it says little as to the extent to which this cognitive skill could be effectively modified in each individual, considering their particular genetic background, by changes in the environment.

As mentioned above, two different types of environmental differences may be distinguished: shared and non-shared environmental features. Shared features of the environment are those aspects of an individual’s environment that are necessarily shared with other people in the family. Examples of these include: general parenting styles and beliefs of the parents; socioeconomic status; the kind of neighborhood where the family lives; and the socio-cultural status of the parents. That is, features of the environment that would be commonly experienced by all children within any particular family. Conversely, non-shared features are any aspect of the environment and any experiences that may be different for different children within the same family; for example, birth order or any random experiences that occur through the lives of the family members (sexual harassment, accidents, infectious illnesses, etc.).

It is also important to note that, depending on the method or methods used to calculate the heritability of a particular trait, the value may differ. This becomes obvious when comparing different work that analyzes the same trait. From the educational perspective, the important message regarding heritability data is not the precise value for any particular trait, but to assume that the cognitive functions relevant for learning are influenced by both the genetics of individuals and their environment, including both shared and non-shared environment.

**FIGURE 1:**
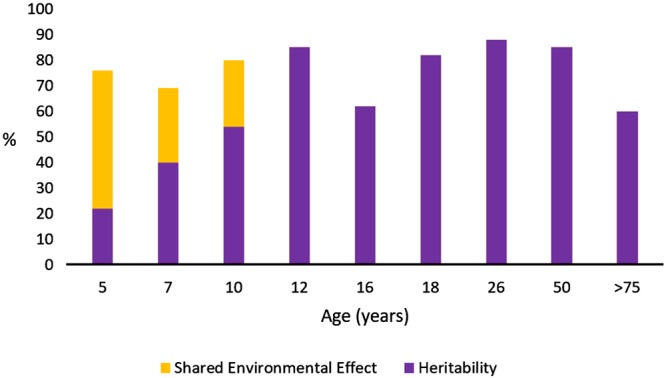
Heritability of intelligence by age in a Dutch cross-sectional twin study. Data from Bouchard (2004). Heritability and shared environmental effects are shown by age; the remainder, bringing the total to 100%, corresponds to environmental effects that are not shared.

As I have already said, heritability is not a constant value, and may vary with age. For example, the heritability of IQ varies from 0.22 in early childhood to more than 0.8 in adulthood, with a substantial effect of shared environment during infancy, but not later in life (Bouchard, 2004; Figure 1). Variations in heritability through the course of people’s lives should be interpreted as percentages. Genetic and environmental effects (including both shared and non-shared environmental effects) must sum 100% (or 1.0 as unitary trait), so an increase of the environmental contribution to the variance implies a corresponding decrease in the relative genetic contribution to the variance of the trait analyzed; and vice versa. In this example, the fact that IQ heritability is significantly lower during infancy could be interpreted as a major effect of educational practices during childhood compared with their effect in adulthood. Finally, there are sex differences in heritability for some particular traits, probably due to the interaction of sex hormones with other genetic networks. For example, Boardman et al. (2008) calculated that the heritability of resilience is 0.52 in males and 0.35 in females, and argued that self-acceptance is one of the most important aspects of psychological functioning that accounts for these differences. Notwithstanding, it must always be kept in mind that heritability does not measure how sensitive a trait might be to a change in environment: even a trait with complete heritability (1.0) might be altered by environmental changes. This fact is crucial for education.

## Heritability of Temperament Traits and Cognitive Functions

A number of papers have been published in which the heritability of psychological traits and cognitive functions is calculated using different populations, tests and methods, including both broad-sense and narrow-sense heritability. Although they do not all coincide in the values given, mainly due to the methods used, they may serve as a basis for comprehending the genetic contribution to brain learning processes, which is the main aim of this review.

Temperament and personality are key elements of psychological traits that may influence learning capacities. Temperament is classically defined as those aspects of personality that express basic emotions such as fear, anger and disgust; and that are developmentally stable and heritable, rather than learned (Shiner et al., 2012). This definition is debated as regards whether to include only procedural learning, which is present in all animals, or also intentional cognitive processes and autobiographical learning, which appear only to be present in humans (Zwir et al., 2018a). It does, however, include personality, which can be defined as the set of psychological traits and mechanisms that form part of the individual, and that are organized, relatively enduring, and influence the individual’s interactions with and adaptations to intrapsychic, physical and social environments (Larsen and Buss, 2017). Most studies have been conducted using the so-called *Big Five* personality traits (extraversion, agreeableness, conscientiousness, neuroticism, and openness) or the *Big Three* (positive emotionality, negative emotionality, and constraint). Bouchard (2004) estimated broad-sense heritability (*H^2^*) for extraversion (0.54), agreeableness (0.42), conscientiousness (0.49), neuroticism (0.48), and openness (0.57) (the *Big Five*); as well as for positive emotionality (0.5), negative emotionality (0.44), and constraint (0.52) (the *Big Three*), with no shared environmental effects. Kandler et al. (2016) gave *h^2^* values for openness of from 0.37 to 0.66, depending on the test used; and for extraversion of from 0.35 to 0.66, also depending on the test. As stated above, although data do not always coincide due to the method used and populations analyzed, they can serve as a basis for understanding the existence of complex genetic contributions to personality.

Other psychological traits which are of interest for learning processes and for which heritability has been calculated, are the so-called Holland’s Six Personality Types, i.e., realistic, investigative, artistic, social, enterprising and conventional. Although the schema has been debated and criticized due to its simplicity (Nauta, 2010), Table 1 summarizes some of the broad-sense heritability data and the effect of shared environment, as reported by Bouchard (2004). Also of potential interest for learning processes, temperamental traits such as positive affect, e.g., attentiveness, and negative affect, e.g., becoming upset, have been described as not constant in an individual but fluctuating over time. According to Zheng et al. (2016), heritability for positive affect is not detectable, while there is a significant shared environmental influence (0.42); although there is a large body of literature on the heritability of positive affect (Wingo et al., 2017). Conversely, negative affect is moderately heritable, as it yields a heritability value of 0.53. Interestingly, both positive and negative affect fluctuate over monthly periods, and these fluctuations show a significant heritability: 0.34 and 0.54, respectively. Similarly, it has been reported that “grit,” defined as perseverance and passion for long-term goals, is a significant predictor of academic success. The heritability of grit has been given as 0.37 (Rimfeld et al., 2016b).

**Table 1:** Estimates of broad-sense heritability and shared environmental effects of Holland’s Six Personality Traits expressed as their contribution to the unitary trait (Bouchard, 2004).

Six Holland Code	Heritability (*H^2^*)	Shared environmental effects
Realistic	0.36	0.12
Investigative	0.36	0.10
Artistic	0.39	0.12
Social	0.37	0.08
Enterprising	0.31	0.11
Conventional	0.38	0.11

Regarding the cognitive functions and other psychological traits directly linked to learning processes and academic success (for example: working memory, rational and experiential thinking, literacy, numeracy, resilience, coping style, attention focus, cognitive control, creativity, musicality, drawing ability, planning capacity, cooperativity, confidence, and relational processing), there is abundant literature available (see Goldberg et al. (2014) for a review of cognitive domain heritability). However, the heterogeneity of the information it contains, which is related to the variety of methods used and populations analyzed (including the age of individuals, and the type of heritability calculated) make it very difficult to systematize. Table 2 summarizes some relevant work on these issues. For simplicity, taking into account that this review is intended to be useful for education professionals and to serve as a basis for understanding genetic contributions to brain learning processes, details of the methods used, population analyzed, and type of heritability are omitted (but they can be found in the papers cited).

**Table 2:** Heritability of some traits linked to learning processes.

Trait	Heritability	Source
Intelligence	from 0.2 to 0.8, depending on age (see Fig. 1) 0.22 to 0.93 depending on test	Bouchard, 2004 Kandler et al., 2016
Extremely high intelligence	0.33	Zabaneh et al., 2018
Creativity	0.08 to 0.62, depending on test	Kandler et al., 2016
Working memory	0.39 0.40–0.65 0.72	Fletcher et al., 2014 Blokland et al., 2011 Hansell et al., 2015
Experimental thinking	0.44	Fletcher et al., 2014
Rational thinking	0.34	Fletcher et al., 2014
Resilience (positive adaptation in the face of adversity) Emotion-oriented coping (as a strategy used to manage adversity) Task-oriented coping (as a strategy used to manage adversity)	0.52 (males) 0.38 (females) 0.14 0.11	Navrady et al., 2018
Attention focus	0.28	Ocklenburg et al., 2016
Cognitive control	0.49	Ocklenburg et al., 2016
Grit (perseverance)	0.37	Rimfeld et al., 2016b
Planning ability	0.53	Tuvblad et al., 2017
Cooperativity	0.13	Hiraishi et al., 2015
Relational processing	0.67	Hansell et al., 2015
Literacy and Numeracy	0.68	Kovas et al., 2013
Musicality	from 0.21 to 0.51, depending on test	Gingras et al., 2015
Drawing ability	0.29	Arden et al., 2014

Individual differences in educational achievement have also been shown to be highly heritable. According to Pokropek and Sikora (2015), exam results show a heritability of 0.57 in mathematics and 0.66 in humanities; while the effect of shared environment is 0.34 in mathematics and 0.30 in humanities, in 9-year-old children. Similarly, the gain in heritability in both mathematics and humanities over the period from 6 to 9 years of age is 0.66. However, in this case the effect of shared environment is 0.0: the effect of non-shared environment is therefore 0.34. This suggests that random experiences, most probably related to educational processes (for example, the particular relations between an individual and their classmates and teachers), have a much greater influence on educational gains than general learning situations. Similarly, Rimfeld et al. (2016a) calculated the heritability of academic achievement in British A-level students to be between 0.35 and 0.75.

It is also important to note that other brain function and structure parameters related to learning processes and academic achievement also show high heritability. For example, effective connectivity in the resting state of the default mode network shows a heritability of 0.54 (Xu et al., 2017). The default mode network, a large-scale brain network of interacting brain regions which is the neurological basis for the self, is known to be involved in many seemingly different functions that are crucial for self-directed learning and other issues related to the self, i.e., autobiographical information, self-reference, a sense of one’s self, theory of the mind, social evaluations, moral reasoning, remembering the past, and thinking about the future.

Similarly, executive functions (as I defined them above: a set of cognitive processes that are necessary for the cognitive control of behavior), i.e., selecting and successfully monitoring behavior that facilitates the attainment of chosen goals, including attentional control, cognitive inhibition, inhibitory control, working memory, and cognitive flexibility, also show moderate to high heritability: from 0.29 to 0.72, depending on the process analyzed (Friedman et al., 2008). As with educational achievement, the effect of shared environment is negligible, but the effect of non-shared environment ranges from 0.24 to 0.71, also depending on the process analyzed.

To summarize, despite most, if not all, traits associated with learning capacity (including temperament and personality, as well as the ability to control behavior toward self-directed goals) show high heritability, environmental factors are also significant. It must always be taken into account that heritability reflects how much of the variance may be attributable to genetic differences within a population but does not measure how sensitive a trait is to a change in the environment. Environmental changes may alter the phenotype of any of these traits. Moreover, in some cognitive functions, the shared environmental factors seem to be highly influential while in others, for example, executive functions, non-shared ones are more influential.

Difficulties in precisely establishing which factors are most influential, together with some common misunderstandings concerning the biological meaning and significance of the concepts of gene function and heritability, have led to some educational proposals allegedly having a scientific basis when in fact they had none at all; as, for example, with the so-called learning styles (Newton, 2015; Macdonald et al., 2017; Newton and Miah, 2017). According to a survey published in by Howard-Jones (2014) more than 90% of education professionals in the United Kingdom, Holland, Turkey, Greece and China thought that the learning process improves when using the appropriate learning style. However, despite the large body of literature on this subject, very little is based on the scientific method, and the results shown by those that are scientific do not allow us to deduce that educational practices based on learning styles are beneficial (Rohrer and Pashler, 2012). For example, a paper published in Doll et al. (2016) on the influence of several alleles of the COMT gene (which as I have said affects dopamine in the prefrontal cortex) as making people learn more quickly from experience when no advice was given, but also making them more readily impressionable when instruction was given, has been interpreted by some as a way to predict learning styles.

Taken together, these data emphasize the importance of both shared learning processes and random experiences (for example, the particular relations between an individual and their classmates and teachers, and any unpredictable experience occurring during their life), which in turn highlights the crucial role of education professionals in maximizing the skills of students to allow them to make the most of their capabilities, through respectful and flexible educational practices, while taking into account the unavoidable genetic influences.

## Linking Environment With Genes

From the data reported above, it is evident that environmental factors shape cognitive functions by affecting the brain. On the one hand, it is known that they affect neural plasticity and contribute to shaping neural networks. For example, we know of the existence of substantial underlying neural plasticity associated with development that supports behavioral changes during adolescence (see Sachser et al., 2018, for a review), as well as during childhood (Miskolczi et al., 2018). Although such neural plasticity is beyond the scope of this review, it is important to highlight its involvement in modifying and adapting behavior through learning. It is also important to note that the connectome (again, the map of neural connections in the brain) which forms through neural plasticity mechanisms, also has genetic influences. Connectome heritability for the whole brain has been calculated to be 0.2, but this depends on particular brain regions (Miranda-Dominguez et al., 2018).

On the other hand, it is also known that a number of environmental factors contribute to regulation of gene functions through epigenetic modifications. This area of “neuroepigenetics” has emerged as an important subfield of neuroscience, linking environmental factors with gene functions that affect cognitive functions (Sweatt, 2013). Epigenetics refers to the reversible regulation of various gene functions, which occurs independently of the DNA sequence, mediated principally by changes in DNA methylation and chromatin structure through post-translational modifications of histones, which are the basic proteins around which DNA is wrapped (Allis, 2015). A number of histone epigenetic modifications have been reported, i.e., acetylation, methylation, phosphorylation, SUMOylation and ubiquitylation, thus establishing complex histone-code-modulating gene expression. Despite the biochemical complexity of these processes, the important thing as far as the scope of this review is concerned is that epigenetic modifications are essential for adaptive long-term regulation of gene expression. In other words, epigenetics allows the linking of environmental particularities with gene function thereby adapting the physiology and behavior of organisms.

It has been reported that DNA methylation contributes, for example, to memory formation and storage, and consequently to learning processes (Day and Sweatt, 2010; reviewed by Schmauss, 2017, and Collins et al., 2019). It has also been demonstrated that differences in DNA methylation influence executive function assessments (Ibrahim et al., 2018), and that early childhood malnutrition is associated with DNA methylations that can impair attention and cognition (Peter et al., 2016). Similarly, epigenetic variance in the gene for the dopamine D2 receptor influences malleability of intelligence (Delvecchio et al., 2016; Kaminski et al., 2018). Although neuroepigenetics is a relatively new field of research, the evidence of its importance in regulating cognitive functions is growing rapidly.

Of special interest are the effects of early childhood traumas and educative environments on cognitive development. For example, it has been reported that childhood abuse correlates with glucocorticoid receptor epigenetic regulation in the brain, favoring the manifestation of depressive behavior later in adolescence and adulthood (McGowan et al., 2009). This specifically affects the hypothalamic-pituitary-adrenal axis (Farrell et al., 2018), as well as the gene for monoamine oxidase A, which in turn shows alleles involved in impulsive behavior and many other effects (Checknita et al., 2018). Similarly, childhood neglect has been reported to correlate with specific epigenetic signatures that have implications for psychiatric vulnerability (Cecil et al., 2016). To cite another case, it has recently been demonstrated that negative parenting (that is, parenting based on little emotional warmth, indifference, neglect, rejection or hostility) correlates with specific epigenetic modifications in a set of genes that can favor depression later in life (Hein et al., 2018).

Despite most of the work focusing on negative experiences and their epigenetic effects on psychiatric vulnerability, changes in epigenetic signatures due to childhood experiences must be seen as an adaptive system that allows people to get over traumas and continue to grow, but which has consequences later in life. This adaptive system of epigenetic signatures is influenced by many kinds of experiences and environmental conditions, without which survival would be much more difficult, and thus it parallels neural plasticity in modifying behavior through learning.

To sum up, the importance of a number of environmental factors for the regulation of gene function that affects personality and cognitive functions, is increasingly being recognized. The work cited above represents only the tip of the iceberg. The purpose of this section is not to review papers linking epigenetics and behavior extensively, but to highlight the importance of learning environments not only for neural plasticity but also for the phenotypic manifestation of the genome, beyond the particular heritability of different learning processes and cognitive functions.

## Conclusion

The most recent genetic and epigenetic data we have available to us emphasize the crucial roles that education professionals, families and society may play when contributing to the education of people who can and want to make the most of their capabilities. These influences can contribute to maximizing the skills that students have available to them to face a changing and uncertain world. Although brain formation and functioning are based on a genetic substrate that influences it to a moderate or high degree, the brain is also malleable and is affected by education and daily experiences, and therefore so too are cognitive functions. As I have previously said, children are certainly no *tabula rasa*, but even a trait with high heritability might be greatly altered by the environment acting directly on brain malleability or through epigenetic modifications. However, it has also to be pointed out that with the current data, it is still not possible to say with a sufficient level of confidence which traits can be improved more easily or which are difficult to change. Not every trait is amenable to meaningful change through educational interventions, and this depends on a variety of factors. Finally, current data also point to another significant factor in education: learning must be perceived as adaptive by the brain, as neural plasticity and epigenetic modifications are, and teaching style is crucial for this perception to occur.

## Author Contributions

The author confirms being the sole contributor of this work and has approved it for publication.

## Conflict of Interest Statement

The author declares that the research was conducted in the absence of any commercial or financial relationships that could be construed as a potential conflict of interest.
